# Minimal B Cell Extrinsic IgG Glycan Modifications of Pro- and Anti-Inflammatory IgG Preparations *in vivo*

**DOI:** 10.3389/fimmu.2019.03024

**Published:** 2020-01-09

**Authors:** Anja Schaffert, Maja Hanić, Mislav Novokmet, Olga Zaytseva, Jasminka Krištić, Anja Lux, Lars Nitschke, Matthias Peipp, Marija Pezer, René Hennig, Erdmann Rapp, Gordan Lauc, Falk Nimmerjahn

**Affiliations:** ^1^Department of Biology, Institute of Genetics, University of Erlangen-Nuremberg, Erlangen, Germany; ^2^Glycoscience Research Laboratory, Genos Ltd., Zagreb, Croatia; ^3^Department of Medicine II, Christian-Albrechts University Kiel and University Medical Center Schleswig-Holstein, Kiel, Germany; ^4^glyXera GmbH, Magdeburg, Germany; ^5^Max Planck Institute for Dynamics of Complex Technical Systems, Magdeburg, Germany; ^6^Faculty of Pharmacy and Biochemistry, University of Zagreb, Zagreb, Croatia

**Keywords:** glycosylation, sialylation, IVIg, IgG, B cells, antibody

## Abstract

Select residues in the biantennary sugar moiety attached to the fragment crystallizable of immunoglobulin G (IgG) antibodies can modulate IgG effector functions. Thus, afucosylated IgG glycovariants have enhanced cytotoxic activity, whereas IgG glycovariants rich in terminal sialic acid residues can trigger anti-inflammatory effects. More recent evidence suggests that terminal α2,6 linked sialic acids can be attached to antibodies post IgG secretion. These findings raise concerns for the use of therapeutic antibodies as they may change their glycosylation status in the patient and hence affect their activity. To investigate to what extent B cell extrinsic sialylation processes modify therapeutic IgG preparations *in vivo*, we analyzed changes in human intravenous IgG (IVIg) sialylation upon injection in mice deficient in B cells or in mice lacking the sialyltransferase 1, which catalyzes the addition of α2,6 linked sialic acid residues. By performing a time course of IgG glycan analysis with HILIC-UPLC-FLR (plus MS) and xCGE-LIF our study suggests that therapeutic IgG glycosylation is stable upon injection *in vivo*. Only a very small fraction of IgG molecules acquired sialic acid structures predominantly in the Fab- but not the Fc-portion upon injection *in vivo*, suggesting that therapeutic antibody glycosylation will remain stable upon injection *in vivo*.

## Introduction

Immunoglobulin G (IgG) antibodies are glycoproteins with pro- and anti-inflammatory effector functions. Thus, binding of IgG to Fcγ receptors (FcγRs) can trigger effector functions such as the release of pro-inflammatory mediators, phagocytosis of opsonized pathogens, or antibody dependent cellular cytotoxicity (ADCC) (1, 2). Apart from host defense, IgG autoantibodies play a major role in tissue destruction and inflammation during autoimmune diseases including the primary Sjögren's syndrome, Systemic Lupus Erythematosus or Rheumatoid Arthritis (3). Moreover, pooled IgG preparations from thousands of donors, called IVIg (intravenous immunoglobulin G), are used as an anti-inflammatory treatment in the therapy of several autoimmune diseases, and during chronic inflammation (4).

The sugar moiety attached to each of two conserved asparagine 297 residues in the constant domain 2 (CH2) of the IgG fragment crystallizable (Fc) is essential for both, the pro- and anti-inflammatory activities of IgG (5) and keeps the IgG molecule in its typical horseshoe shape critical for FcγR binding (6–8). Apart from IgG Fc-glycosylation, about 10–15% of serum IgG contains an N-linked sugar moiety in the IgG fragment antigen binding (Fab) domain, which is generated *de novo* during the process of IgG hypermutation (9, 10). More recent evidence suggests that IgG Fab glycosylation helps to regulate IgG specificity (11). With respect to IVIg, especially terminal sialic acid residues were shown to be responsible for its anti-inflammatory activity. Thus, while desialylated IVIg lost its capacity to suppress a wide variety of autoimmune diseases in mice (12, 13), IVIg preparations enriched for terminal sialic acid residues showed an enhanced anti-inflammatory activity (5, 14). Of note, enriching cytotoxic antibodies for terminal sialic acid residues decreased their activity *in vivo* and *in vitro* in some but not all studies (15). Consistent with this reduced activity a reduction in affinity of highly sialylated IgGs for select activating FcγRs was noted (5). Due to the potent immune modulating functions of select IgG glycoforms, new therapeutic approaches try to alter IgG activity by modulating its glycosylation *ex vivo* (5, 16), or by changing the glycosylation status in the patient by enzymatic approaches (17–19).

Due to the potent immunomodulatory activity of the IgG sugar moiety, a precise monitoring of therapeutic IgG glycosylation has become standard before using new recombinant antibody preparations or consecutive batches of already approved antibodies in patients. This in-depth characterization relies on the fact that once an IgG antibody is injected into the patient, the sugar structures remain stable and are not subject to *in vivo* processing. However, recent studies suggest that terminal α2,6-linked sialic acids may be attached independently of the B cell secretory pathway (20, 21). According to these results, B cell independent IgG sialylation is achieved in the liver by secreted ST6Gal1 produced by cells lining the liver central veins. As a sugar donor, CMP-sialic acid at least partially derived from degranulating platelets may be used. More recently, it was suggested that in addition to antibodies the surface of cells may also become sialylated through this process (22). With respect to therapeutic antibody preparations, these findings raise the severe concern, that this process may alter the activity of therapeutic antibodies in the patient. Thus, cytotoxic antibodies may become less active due to decreased binding to activating FcγRs, while intravenous IgG preparation may become more active and may in the worst-case lead to an unwanted strong immune suppression. Moreover, the genetic heterogeneity of the human population and age dependent alterations of immune responses may further complicate to predict how stable therapeutic antibodies are in individual patients and with respect to glycosylation.

To address this issue and identify to what extent therapeutic IgG preparations are subject to B cell independent sialylation, we made use of two mouse strains lacking either B cells or ST6Gal1, which is the responsible enzyme for adding terminal sialic acid residues to the IgG sugar moiety (23–25). Both mouse strains were injected with human IVIg preparations having either a normal or strongly reduced level of sialylated IgG glycoforms. IgG N-glycan analysis by HILIC-UPLC-FLR (plus MS-detection) and xCGE-LIF of mouse serum for several consecutive days after IVIg administration revealed that IVIg glycosylation is very stable upon injection *in vivo*. Only a very small fraction, predominantly of disialylated IgG N-glycans attached to the Fab- rather than the IgG Fc-portion appeared to increase over time. In summary, our results suggest that B cell extrinsic IgG sialylation may not modulate therapeutic IgG activity post injection *in vivo*.

## Materials and Methods

### Mice

C57BL/6 mice were bought from Janvier, μMT^−/−^ mice were obtained from The Jackson Laboratory. Rag2/γc/FcεRγ/FcγR2b^−/−^ were generated by breeding Rag2/γc/FcεRγ/FcγR2b^−/−^ mice (26) with FcγR2b^−/−^ mice. ST6Gal1^−/−^ mice were kindly provided by Prof. Dr. rer. nat. Lars Nitschke (FAU Erlangen, Germany). Rag2/γc/FcεRγ/FcγR2b^−/−^, μMT^−/−^, and ST6Gal1^−/−^ mice were kept in the animal facilities of Friedrich-Alexander-University Erlangen-Nuremberg under specific pathogen-free conditions in individually ventilated cages, in accordance with the guidelines of the NIH and the legal requirements of Germany and the USA. All animal experiments conducted in the animal facility of the FAU were approved by government of Lower Franconia.

### Reagents

Human IVIg was purchased from Biotest AG (Intratect, 100 mg/ml). α2-3,6,8 neuraminidase was purchased from New England BioLabs (P0720L, 50,000 U/ml) and used at a concentration of 0,04 U/μg IVIg. Rituximab (MabThera®, Roche) was kindly provided by Prof. Dr. rer. nat Matthias Peipp (Christian-Albrechts-University Kiel, Germany). TA99-mIgG2c was purchased from BioXcell (USA),

### Fluorescence-Activated Cell Sorting Analysis

Murine blood was obtained from the retroorbital plexus and erythrocytes were lysed. To reduce unspecific binding to Fc receptors, cells were incubated on ice for at least 10 min with Fc-block (clone 2.4G2, 10 μg/ml). Cells were washed and incubated on ice for at least 15 min with combinations of the following antibodies: APC-Fire750-conjugated CD45 (clone 30-F11), BV510-conjugated CD11b (clone M1/70), and PE-conjugated NKp46 (clone 29A1.4), PE-conjugated Ly6G (clone 1A8), PE-conjugated TCRβ (clone H57-597), and PE-Cy7-conjugated CD19 (clone 6D5) (all purchased from Biolegend). FITC-conjugated SNA was purchased from Vector Laboratories. Analysis was restricted to viable cells, which were identified by exclusion of cells positive for the nucleic acid binding dye 4'6-diamino-2-phenylindol (DAPI). Experiments were acquired on a FACS Canto II (BD) and analyzed using FACS Diva software (BD).

### ELISA

Sera of 8–9 week old C57BL/6, μMT and ST6Gal1^−/−^ mice were collected and stored at−20°C until further use. For quantification of total serum IgM and IgG the Mouse IgM ELISA Quantification Kit and the Mouse IgG ELISA Quantification Kit (Bethyl) were used according to the manufacturer's instructions: ELISA plates were coated with 100 ng/well goat anti-mouse IgM or IgG in Carbonate/Bicarbonate for 1 h at room temperature. After washing unspecific binding was blocked with PBS/1% BSA for 1 h at room temperature. Sera were diluted 1:2,500 (IgM) or 1:10,000 (IgG) in PBS/1% BSA and incubated for 1 h at room temperature and, after adequate washing, bound IgM and IgG antibodies were detected by 1:20,000 diluted (in PBS/1% BSA) anti-IgM-HRP or anti-IgG-HRP antibody in PBS/1% BSA (incubated for 1 h at room temperature). For detection, TMB Solution was added and the reaction was stopped with 6% orthophosphoric acid. OD was measured with VersaMax tunable microplate reader (Molecular Devices) at 450 and 650 nm. For detection of remaining human IVIg or neuraminidase treated IVIg in the sera of ST6Gal1^−/−^ mice the Human IgG ELISA Quantification Kit (Bethyl) was used according to the above-described protocol. ELISA plates were coated with 100 ng/well goat anti-human IgG. Sera were diluted 1:50,000.

### *In vivo* Experiments

10 mg IVIg (Intratect, Biotest, Germany), 10 mg neuraminidase treated IVIg (NeuIVIg) or 1 mg of the murine antibody TA99-mIgG2c (BioXcell, USA), which is directed against the glycoprotein 75 (gp75), was injected into eight- to nine-week old μMT or ST6Gal1^−/−^ mice. Two, four and six days after injection sera were collected and analyzed by HILIC-UPLC-FLR (hydrophilic interaction ultra performance liquid chromatography with fluorescence detection; IVIg and NeuIVIg treated serum samples) or xCGE-LIF (multiplexed capillary gel electrophoresis with laser-induced fluorescence detection; IVIg, NeuIVIg and TA99 treated serum samples) to analyze IgG specific glycan structures.

### Rituximab-IgG-Induced B Cell Depletion in PBMC Humanized Rag2/γc/FcεRγ/FcγR2b^−/−^

PBMCs were isolated by density centrifugation from individual buffy coats. Isolated PBMCs were frozen and stored in liquid nitrogen until further use. Adult Rag2/γc/FcεRγ/FcγR2b^−/−^ mice were irradiated with 6 Gy and injected intraperitoneally with 1 × 10^7^ human peripheral blood mononuclear cells (PBMCs) 6 h after irradiation as described previously (27). Eighteen hours after PBMC transfer, an equal amount of 0.5 μg anti-CD20 rituximab IgG1 (MabThera®) in the serum of rituximab injected mice was given intraperitoneally and 24 h later B cell counts in the peritoneum were analyzed by flow cytometry.

### Glycan Analysis

#### IgG Isolation

The IgG was isolated using protein G monolithic plates (BIA Separations, Ajdovščina, Slovenia) as described previously (28). Briefly, 100–400 μl of serum was diluted in ratio 1:7 with 1 × PBS, pH 7.4 and filtered through 0.45 μm GHP filter plate (Pall Corporation, Ann Arbor, MI, USA). After filtration, serum samples were applied to the protein G plate and instantly washed with 1 × PBS, pH 7.4, to remove unbound proteins. IgGs were eluted with 1 ml of 0.1 M formic acid (Merck, Darmstadt, Germany) and neutralized with 1 M ammonium bicarbonate (Merck, Darmstadt, Germany).

### Sample Preparation for HILIC-UPLC-FLR Analysis of Glycans

#### Methanol Desalting

Volumes of eluates corresponding to 100 μg of IgG were dried in a vacuum concentrator. Samples were then desalted by methanol precipitation. For methanol desalting 1 mL of cold (−20°C) methanol (MeOH) was added to each sample and resuspended. The plate containing samples was then closed with adhesive seal and centrifuged at 2,200 g for 15 min. After centrifugation, 970 μL of MeOH was discarded and 1 ml of cold MeOH was added again to each sample and resuspended. After that, the plate was again centrifuged at 2,200 g for 15 min. After a second centrifugation, the 970 μL of MeOH again was discarded and the remaining sample was then dried by vacuum centrifugation.

#### Glycan Release

The dried, desalted samples were dissolved in 30 μL 1.33% SDS (w/v) (Invitrogen, Carlsbad, CA, USA) and denatured by incubation at 65°C for 10 min. After incubation, samples were left to cool down to room temperature for 30 min. Subsequently, 10 μL of 4% Igepal-CA630 (Sigma-Aldrich, St. Louis, MO, USA) was added to the samples and incubated on a shaker for 15 min. After shaking, 1.2 U of PNGase F (Promega, Madison, WI, USA) in 10 μL 5 × PBS were added and incubated overnight at 37°C for N-glycan release.

#### Glycan Labeling

The released N-glycans were labeled with 2-aminobenzamide (2-AB). The labeling mixture was freshly prepared by dissolving 2-AB (Sigma-Aldrich, St. Louis, MO, USA) in DMSO (Sigma-Aldrich, St. Louis, MO, USA) and glacial acetic acid (Merck, Darmstadt, Germany) mixture (70:30, v/v) and by adding 2-picoline borane (Sigma-Aldrich, St. Louis, MO, USA) to a final concentration of 19.2 mg/mL for 2-AB and 44.8 mg/ml for 2-picoline borane. A volume of 25 μL of labeling mixture was added to each N-glycan sample in the 96-well plate and the plate was sealed using adhesive tape. Samples were mixed by a 10 min shaking step, followed by 2 h incubation at 65°C. After incubation, samples were left to cool down to room temperature for 30 min.

#### HILIC-SPE

The samples (in a volume of 75 μL) were mixed with 700 μL of cold 100% ACN (Sigma-Aldrich, St. Louis, MO, USA). Free label and reducing agent were removed from the samples using HILIC-SPE on a 0.2 μm GHP filter plate (Pall Corporation, Ann Arbor, MI, USA). Solvent was removed by application of vacuum using a vacuum manifold (Millipore Corporation, Billerica, MA, USA). All wells were prewashed using 200 μL of 70% ethanol (Carlo Erba Reagents, Val de Reuil, France), followed by 200 μL water and equilibrated with 200 μL of cold 96% ACN. The samples were loaded onto GHP filter plate and incubated for 2 min before the vacuum application. The wells were subsequently washed 5 × using 200 μL of cold 96% ACN. The last washing step was followed by centrifugation at 165 × *g* for 5 min. Glycans were eluted two times with 90 μL of ultrapure water after 15 min of shaking at room temperature followed by centrifugation at 165 × *g* for 5 min. The combined eluates were stored at −20°C until usage.

#### HILIC-UPLC-FLR Analysis of IgG Glycans

Fluorescently labeled N-glycans were separated by HILIC on a Waters Acquity UPLC instrument (Milford, MA, USA) consisting of a quaternary solvent manager, sample manager and a fluorescence (FLR) detector set with excitation and emission wavelengths of 250 and 428 nm, respectively. The instrument was under the control of Empower 3 software, build 3471 (Waters, Milford, MA, USA). The UPLC-FLR system was equipped with a hydrophilic interaction liquid chromatography (HILIC) column, a Waters BEH Glycan chromatography column (100 × 2.1 mm i.d., 1.7 μm BEH particles). The separation used a gradient of 75% solvent B (100% ACN; solvent A: 100 mM ammonium formate pH 4.4) to 62% solvent B over 27 min, with a flow of 0.4 ml/min. Solvent B was maintained at 62% for an additional 5 min. The column was then washed for 2 min with 100% of solvent A. Initial conditions were restored in 1 min and held for an additional 5 min to ensure column re-equilibration. Samples were maintained at 10°C before injection, and the separation temperature was 60°C. The system was calibrated using an external standard of hydrolyzed and 2-AB labeled glucose oligomers from which the retention times for the individual glycans were converted to glucose units (GU). The chromatographic glycan peaks resulting from the HILIC-UPLC-FLR analysis were integrated using an automatic processing method with a traditional integration algorithm after which each chromatogram was manually corrected to maintain the same intervals of integration for all samples. The amount of glycans in each peak was expressed as % of total integrated area. Peak annotation of human and murine IgG was performed according to Pučić et al. (28) and Kristic et al. (29).

#### HILIC-UPLC-FLR-MS/MS

2AB labeled N-glycans were separated and measured on an Acquity UPLC H-class instrument coupled to Compact Q-TOF mass spectrometer via Ion Booster ion source. Both instruments were operated under HyStar software version 3.2 (Bruker Daltonics). N-glycans were separated on a Waters BEH Glycan chromatography column, 100 × 2.1 mm, 1.7 μm BEH particles, using 100 mM ammonium formate, pH 4.4., as solvent A and acetonitrile as solvent B. Solvent A was prepared by diluting 2 M stock solution of ammonium formate, pH 4.4 with ultrapure water. For separation linear gradient of 25–38% of solvent A at the flow rate of 0.4 mL/min in a 32 min analytical run was used. Sample was maintained at 10°C before injection, while the separation temperature was 60°C. Fluorescent detector was set with excitation and emission wavelengths of 250 and 428 nm, respectively. Data processing was performed using an automatic processing method with a traditional integration algorithm after which each chromatogram was manually corrected to maintain the same intervals of integration for all the samples. Relative abundance of each obtained peak was expressed as percentage of total integrated area. Mass spectrometer was operated in a positive ion mode with capillary voltage set to 2,250 V and nebulizing gas at pressure of 5.5 Bar. Drying gas (nitrogen) was applied to source at a flow rate of 4 L/min and temperature of 300°C, while vaporizer temperature was set to 300°C and flow rate of 5 L/min. Nitrogen was used as a source gas, while argon was used as collision gas. Ion energy was set to 4 eV, transfer time was 100 μs. Spectra were recorded in *m*/*z* range of 50–3,000 at a 0.5 Hz frequency. N-glycan structures were assigned based on retention time, measured mass and fragmentation spectra using GlycoMod (30) (http://web.expasy.org/glycomod/) and GlycoWorkbench (31).

### Sample Preparation for xCGE-LIF Analysis of Glycans

#### Preparation of Fc and Fab Fractions of Antibodies

IgG-Fc beads (6 μL; CaptureSelect™ IgG-Fc (ms) Affinity Matrix, Thermo Scientific, USA) were dispensed into each well of Orochem filter plate (Orochem Technologies Inc., USA) in order to capture antibodies through their Fc region and washed three times with 200 μl 1 × PBS (pH 7.4) on a vacuum manifold. Eluates obtained after isolation on Protein G plate were added to each well of the Orochem filter plate in a volume ranging from 200 to 400 μL (~90 μg of antibody) and incubated for 1 h at room temperature. Samples were washed four times with 200 μL 1 × PBS (pH 7.4) to remove unbound antibodies. The recombinant streptococcal IdeS enzyme (FabRICATOR, Genovis, Lund, Sweden; 1 U per 1 μg of protein) was combined with 35 μL 1 × PBS (pH 6.6) and added to each sample. Samples were incubated in a humid chamber at 37°C for 18 h. Fab fractions were collected by centrifugation for 2 min at 50 × *g* in a PCR plate (Thermo Fischer Scientific, MA, USA). Remaining Fc fractions were washed three times with 200 μl 1 × PBS (pH 7.4) and additional three times with 200 μl ultrapure water. For elution of Fc fragments, 100 μL od 0.1 M formic acid (pH 2.5) was added to each well of Orochem filter plate and neutralized with 17 μL 1 M ammonium bicarbonate. Fc fragments were collected in a clean PCR plate and neutralized with 17 μL 1 M ammonium bicarbonate.

#### Methanol Desalting

Volumes of IgG eluates corresponding from 3 to 10 μg of IgG were dried in a vacuum concentrator and desalted following previously described protocol for methanol precipitation.

#### N-Glycan Release

The dried, desalted samples were dissolved in 3 μL of 1.66 × PBS with 4 μL 0.5% SDS (w/v) (Invitrogen, Carlsbad, CA, USA) and denatured by incubation at 65°C for 10 min. After incubation, 2 μL of 4% Igepal-CA630 (Sigma-Aldrich, St. Louis, MO, USA) was added to the samples and incubated on a shaker for 15 min. After shaking, 1.2 U of PNGase F (Promega, Madison, WI, USA) in 1 μL 5 × PBS was added and incubated for 3 h at 37°C for N-glycan release. Samples were dried in a vacuum concentrator afterward.

#### Glycan Labeling and HILIC-SPE

Dried samples were labeled with 8-aminopyrene-1,3,6-trisulfonic acid (APTS, Sigma-Aldrich, St. Louis, MO, USA) and cleaned by HILIC-SPE on BioGel P10 (Bio-Rad, Hercules, CA, USA) in 96-well format as described previously (32).

#### Multiplexed Capillary Gel Electrophoresis With Laser-Induced Fluorescence (xCGE-LIF)

First xCGE-LIF measurement was carried out without internal normalization standard to visually assess signal intensities in the samples. Reaction mixture consisted of 3 μl of N-glycan post-cleanup eluate, 1 μl GeneScan 500 LIZ Size Standard (Applied Biosystems, Foster City, CA, USA; 1:50 dilution in Hi-Di Formamide) and 6 μl Hi-Di Formamide (Applied Biosystems, Foster City, CA, USA) pipetted into MicroAmp Optical 96-well Reaction Plate (Applied Biosystems, Foster City, CA, USA), sealed with a 96-well plate septa (Applied Biosystems, Foster City, CA, USA) and briefly centrifuged to avoid air bubbles at the bottom of the wells. Second measurement was also carried out in total volume of 10 μl and contained 3 μl of N-glycan post-cleanup eluate (depending on the signal intensity in the first xCGE-LIF run), 1 μl GeneScan 500 LIZ Size Standard (1:50 dilution in Hi-Di Formamide), 1 μl of NormMix (glyXera, Magdeburg, Germany) in Hi-Di Formamide. The xCGE-LIF measurement was performed in a 3130 Genetic Analyzer (Applied Biosystems, Foster City, CA, USA), equipped with a 50 cm 4-capillary array filled with POP-7 polymer (Applied Biosystems, Foster City, CA, USA). Electrokinetic sample injection was performed at 7.5–15 kV for 5 or 10 s depending on the signal intensity; samples were analyzed with a running voltage of 15 kV and run time of 3,400 s. Raw data files were converted to.xml file format using DataFileConverter (Applied Biosystems, Foster City, CA, USA) and analyzed using the glycan analysis tool glyXtool™ (glyXera, Magdeburg, Germany). GlyXtool™ software was used for structural identification by patented migration time normalization to an internal standard and N-glycan database driven peak annotation, for data comparison and for integration of normalized peak heights (33, 34).

### Statistical Analysis

The statistical significance of the data was determined as indicated in the figure legends. In brief, the Kruskal-Wallis test, followed by Dunn‘s multiple comparison test, or repeated measures two-way ANOVA with Bonferroni post-test were used to determine statistical differences between more than two groups. To indicate different levels of significance, a *p* 0.05 was assigned one asterisk, a value smaller than 0.05 but larger than 0.001 was assigned two asterisks and a value smaller than 0.001 was assigned three asterisks.

## Results

### Model System to Study B Cell Independent Sialylation of Therapeutic IgG

**Figure 1:**
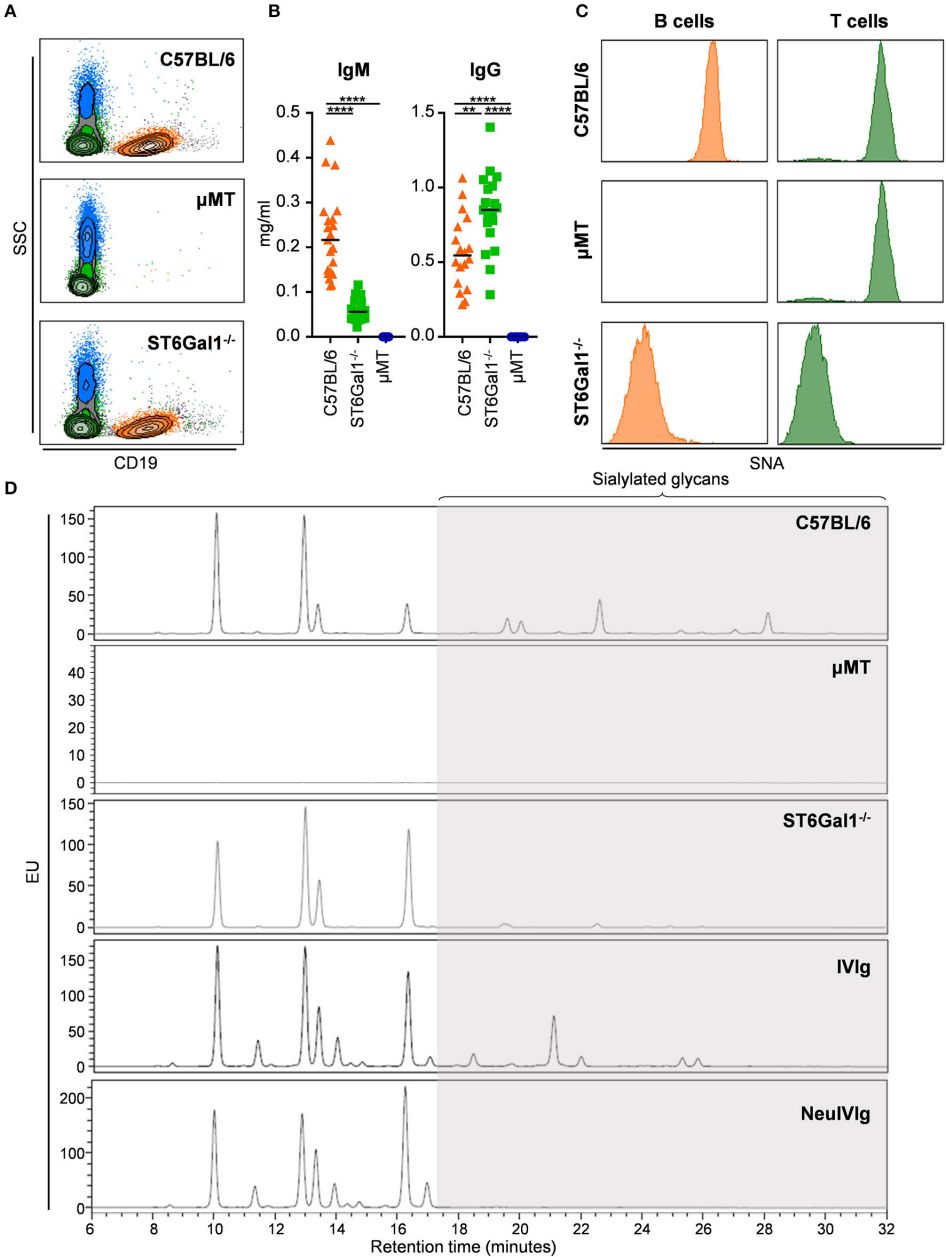
Characterization of the experimental system to study extrinsic IgG sialylation. **(A–C)** C57BL/6, μMT, and ST6Gal1^−/−^ mice were analyzed for the presence of B cells in the blood by FACS analysis **(A)**, serum IgM and IgG by ELISA **(B)** as well as for the presence of a2,6-linked sialic acid residues on the surface of B and T cells by staining with sambucus nigra agglutinin (SNA). **(D)** Shown are representative glycoanalysis of serum IgG from the indicated mouse strains and of IVIg or neuraminidase treated IVIg (NeuIVIg) by HILIC-UPLC-FLR. Statistical significance was evaluated with a one-way ANOVA test and a Bonferroni correction. **p = 0.01, ****p = 0.0001. Horizontal lines in **(B)** represent mean values (*n* = 10–20). SSC, side scatter; EU, emission units.

To determine to what extent therapeutic IgG preparations are subject to B cell independent sialylation, we made use of two mouse strains, namely ST6Gal1 deficient (ST6Gal1^−/−^) and μMT mice. While ST6Gal1^−/−^ mice lack the sialyltransferase 1, catalyzing the addition of α2,6 linked sialic acid residues (23, 35), μMT mice have a disruption of the gene encoding the μ-chain constant region resulting in an arrest of B cell development at the pre-B-cell stage (36). As shown in Figure 1, we started with a characterization of the two *in vivo* model systems with respect to B cell development and the presence of a2,6-linked sialic acid residues. As expected, μMT mice had no B cells in the blood, while the amount of B cells of ST6Gal1^−/−^ mice was comparable to C57BL/6 mice (Figure 1A). Consistent with the absence of B cells in μMT mice, no IgM and IgG antibodies were detectable in the serum (Figure 1B). In contrast, ST6Gal1^−/−^ mice showed normal levels of B cells, strongly reduced levels of serum IgM and a trend toward higher levels of serum IgG as described before (Figure 1B) (35, 37). Staining with SNA (sambucus nigra agglutinin)—a plant lectin specifically detecting α2,6 linked sialic acids—demonstrated that ST6Gal1^−/−^ mice were negative for SNA on B and T cells, whereas μMT mice showed a T cell SNA staining pattern comparable to C57BL/6 mice (Figure 1C). Finally, the serum IgG glycosylation pattern of C57BL/6, μMT and ST6Gal1^−/−^ mice was analyzed by HILIC-UPLC-FLR confirming the absence of a2,6 linked sialic acid species on serum IgG of ST6Gal1^−/−^ mice (Figure 1D). In addition, we analyzed the sugar structures present in IVIg and neuraminidase digested IVIg preparations (NeuIVIg), which were used for studying B cell independent sialylation *in vivo* (Figure 1D). As expected, no sialic acid containing sugar moieties were detectable in neuraminidase digested IVIg, allowing a detection of extrinsic *de novo* sialylation *in vivo* with the greatest possible sensitivity.

### Analysis of B Cell Extrinsic IVIg Sialylation *in vivo*

**Figure 2:**
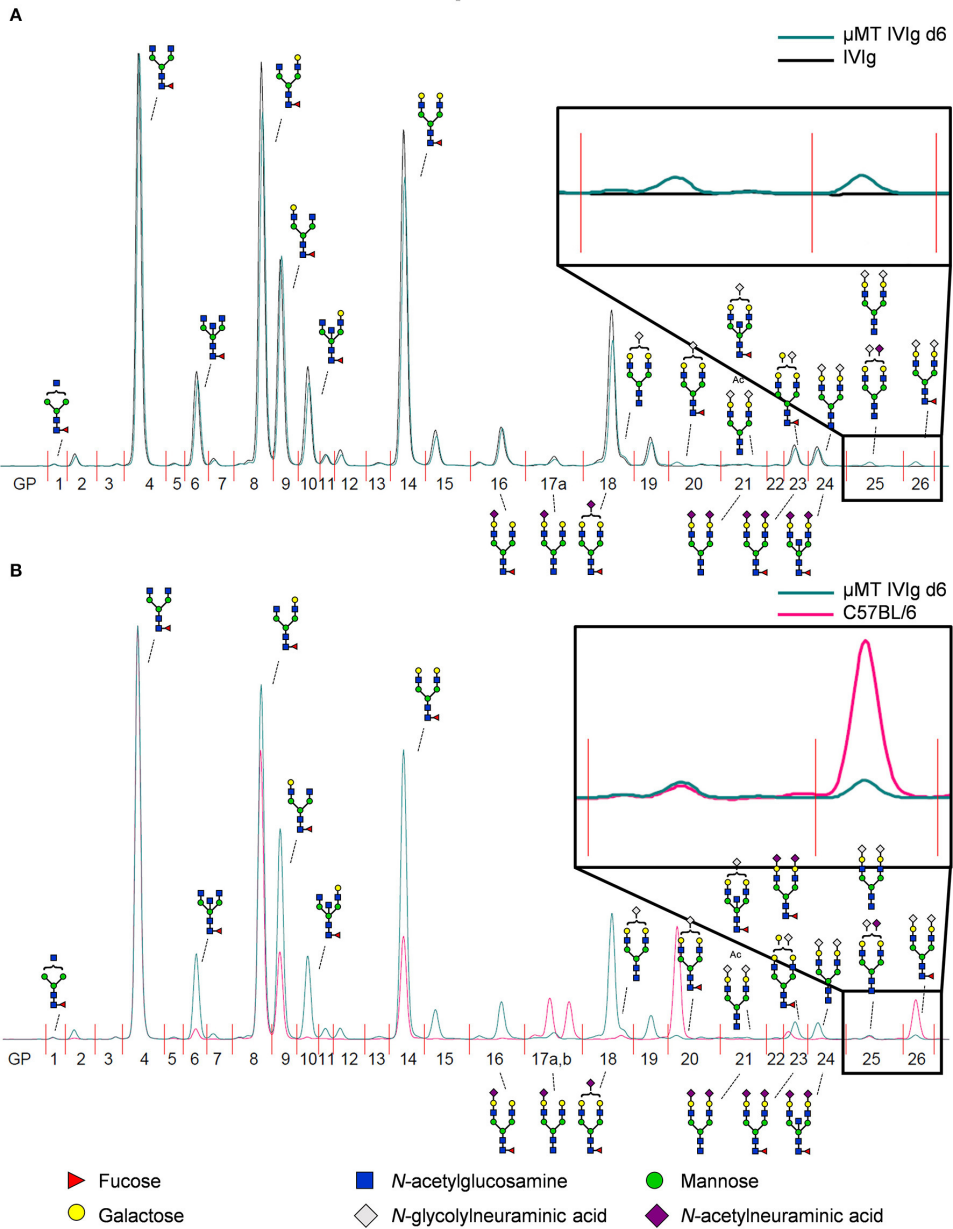
Detection of *de novo* sialylated IgG glycoforms *in vivo*. **(A)** Shown is an overlay of a representative HILIC-UPLC-FLR analysis of IVIg before and 6 days after injection into μMT mice (μMT IVIg d6). **(B)** Depicted is an overlay of a HILIC-UPLC-FLR analysis of mouse serum IgG and IVIg 6 days post injection into μMT mice. Enlarged insets highlight additional glycan peaks (GP) that were not present in the original IVIg preparation. Schematic drawings of the most prominent sugar structures (mouse structures on top, human structures on the bottom) are assigned to the respective peaks. Legend at the bottom of the figure explains the symbols used for individual sugar structures. See also Figure S2 for additional peak information.

To address if therapeutic IgG preparations are subject to B cell extrinsic *de novo* sialylation *in vivo*, we injected 10 mg IVIg in B cell deficient μMT mice and analyzed alterations in IVIg glycosylation in the serum of these animals by hydrophilic interaction ultra-performance liquid chromatography with fluorescence detection (HILIC-UPLC-FLR). In addition, the identity of each peak, were sialylation was expected (GP15–26), was confirmed by mass spectrometry (fragmentation spectra see Figure S1). As mouse IgG glycans terminate with N-glycolylneuraminic acid (Neu5Gc), while human IgGs carry terminal N-acetylneuraminic acids (Neu5Ac), the injection of human IgG into mice allows an unequivocal detection of B cell independent sialylation (29, 38–40). An overview of all detected murine and human glycan structures is shown in Figure S2. When we compared the HILIC-UPLC-FLR profiles of IVIg before and 6 days after injection in μMT mice we found that the IVIg glycosylation profiles overlapped almost completely (Figure 2A). However, some small additional glycan peaks (GP20, GP25, GP26) were detected that were not present in the original IVIg preparation (enlarged inset in Figure 2A). Interestingly all of those novel glycan peaks overlapped with sialic acid containing sugar structures (GP20 containing one sialic acid residue, GP25 and GP26 containing two sialic acid residues with (GP26) or without (GP25) fucose) selectively present in serum IgG from C57BL/6 mice (Figure 2B), suggesting that a very small level of extrinsic sialylation of IVIg can occur in mice.

**Figure 3:**
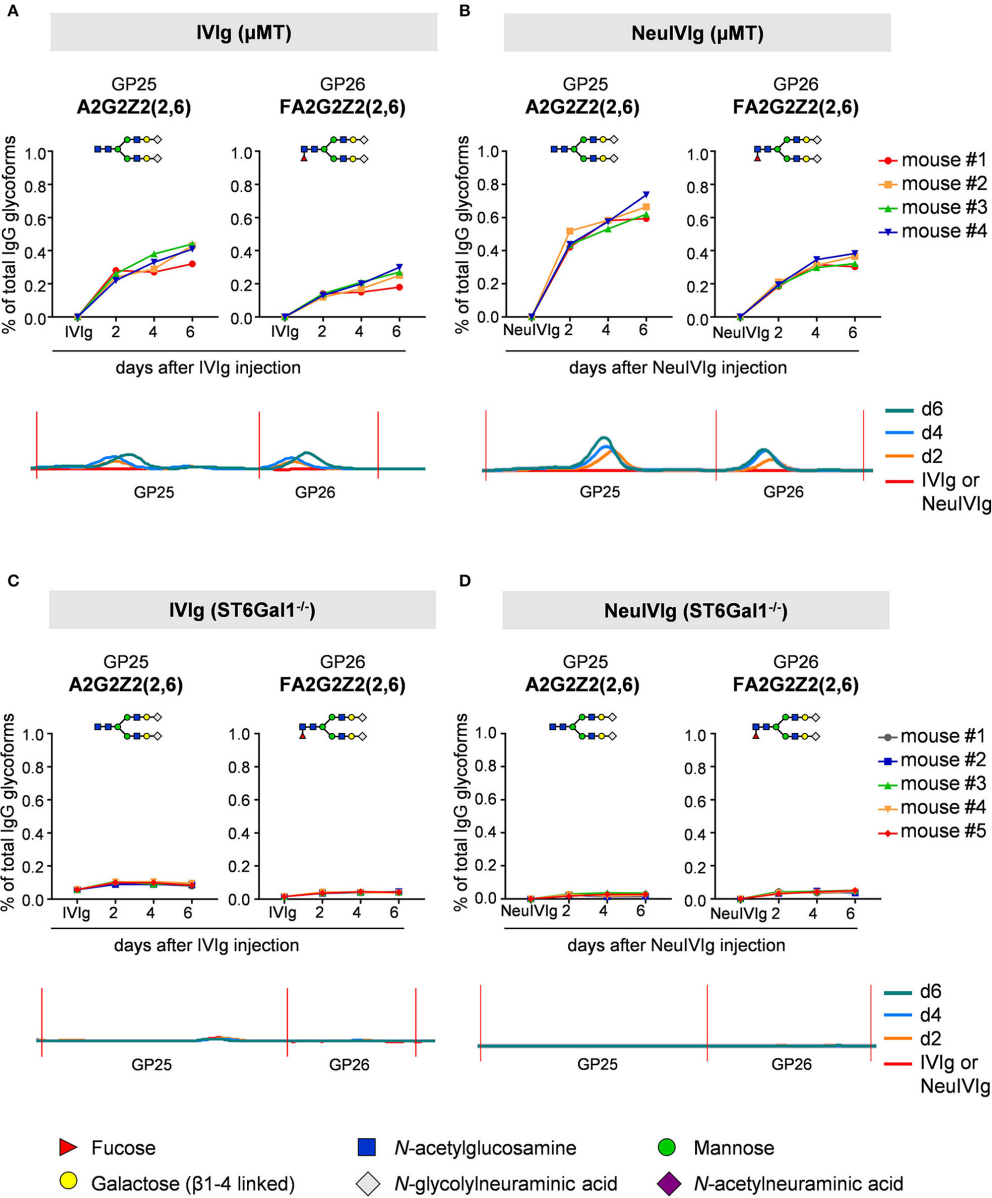
Kinetics of extrinsic IgG sialylation in μMT and ST6Gal1 deficient mice. Shown are the relative changes (% of total IgG glycoforms in the preparation) in glycopeaks (GP) 25 and 26 in IVIg **(A,C)** or neuraminidase treated IVIg **(B,D)** before (IVIg or NeuIVIg) and 2, 4, and 6 days after injection into μMT **(A,B)** or ST6Gal1 deficient mice **(C,D)** by HILIC-UPLC-FLR analysis. Changes in the respective glycopeak in individual mice are shown at the top of each figure (*n* = 4–5). The bottom of each Figure shows a representative HILIC-UPLC-FLR trace of GP25 and 26. In each subfigure a schematic drawing of the sugar structure for GP25 and 26 is depicted. The Figure legend at the bottom explains the symbols used for the schematic representation of the sugar moieties.

**Figure 4:**
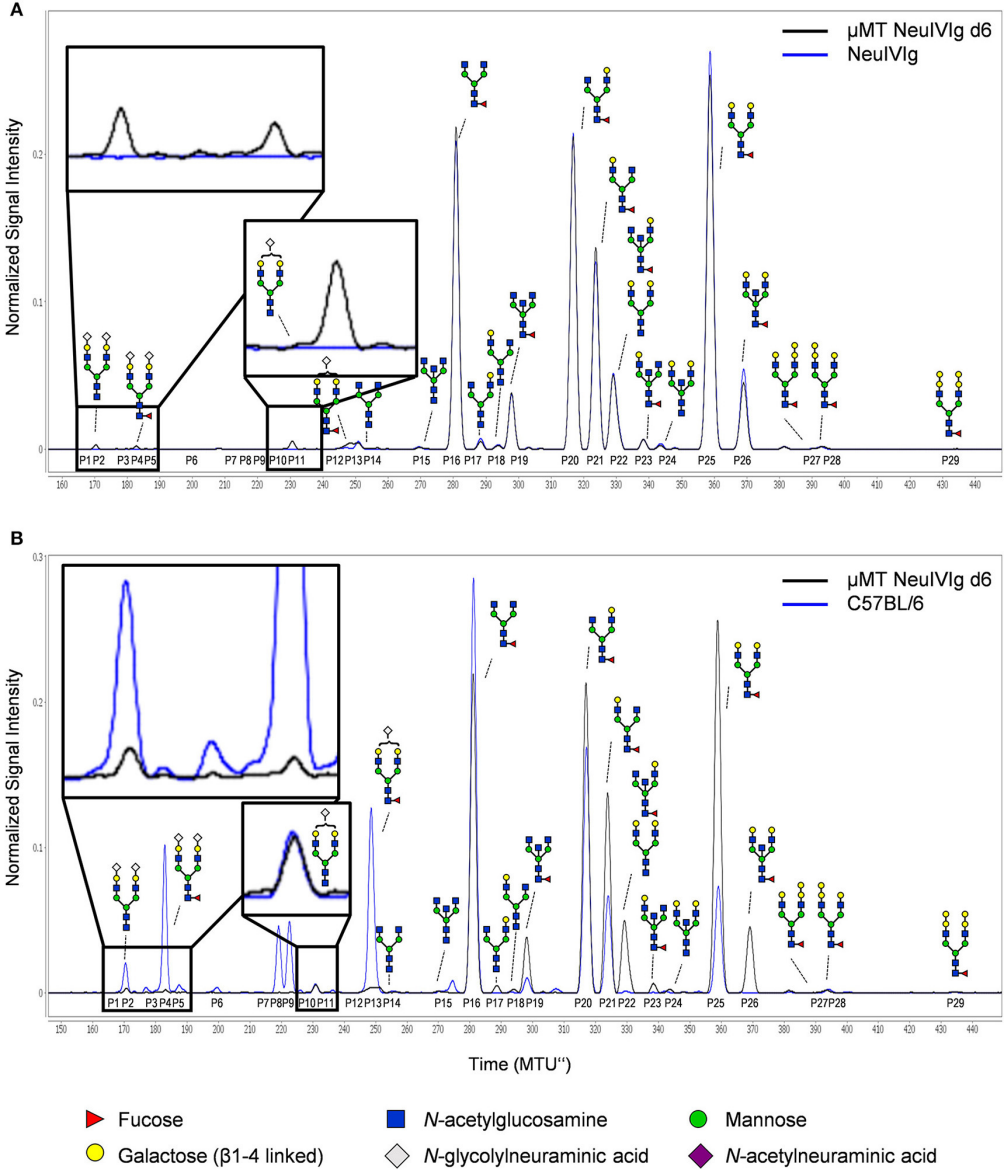
Detection of *de novo* sialylation of human IgG *in vivo* by xCGE-LIF. **(A)** Depicted are representative overlays of xCGE-LIF IgG glycoanalysis of neuraminidase treated IVIg (NeuIVIg) before and 6 days after injection into μMT mice (μMT NeuIVIg d6). **(B)** Shown are representative overlays of xCGE-LIF glycoanalysis of serum IgG from C57BL/6 mice or from μMT mice 6 days post injection with neuraminidase treated IVIg. Schematic drawings of the sugar moieties are depicted for the major peaks. Enlarged insets emphasize peaks detected selectively on NeuIVIg 6 days post injection into μMT mice. The Figure legend at the bottom of the Figure depicts the symbols used for individual sugar residues. See also Figure S2 for additional peak information. MTU”, normalized migration time units.

We next analyzed the kinetics of B cell independent IgG sialylation by studying changes in IVIg sialylation 2, 4, and 6 days after injection into μMT mice (Figure 3 and Figure S3). As some studies suggest, that the level of IgG sialylation may affect antibody half-life and FcRn binding (41, 42), we first assessed if both, sialylated and non-sialylated IVIg preparations had a comparable half-life. As shown in Figure S3A, however, we noted no effect in the half-life of sialylated and asialylated IVIg (5), ensuring that both IVIg preparations had a comparable chance to become resialylated *in vivo*. Indeed, the presence of both disialylated sugar structures (GP25 and 26) slowly increased over time (Figure 3A). To increase the amount of acceptor sites for extrinsic IgG sialylation we also injected IVIg pretreated with neuraminidase (NeuIVIg). As shown in Figure 3B, however, this only mildly increased the level of extrinsic IgG sialylation on afucosylated sugar moieties, despite the availability of large amounts of IgG-G2 glycosylation variants. In total, only 1% of IgG glycovariants present in NeuIVIg acquired a disialylated sugar moiety 6 days after injection into μMT mice. To further validate these results, we repeated this experiment in ST6Gal1^−/−^ mice, which are not able to add α2,6-linked sialic acid residues and therefore served as a negative control. As shown in Figures 3C,D and Figure S3, the sialylation of IVIg or neuraminidase digested IVIg did not change over time in these animals, suggesting that the increase in GP25 and GP26 were indeed due to *de novo* IgG sialylation by ST6Gal1. In contrast, the small increase in monosialylated fucosylated IgG glycostructures (GP20) was also evident in ST6Gal1 deficient mice (Figures S3B,C). Moreover, afucosylated monosialylated glycan forms (GP18, A2G2Z1) of IVIg or NeuIVIg (Figure 4B) did not increase over time (Figure S3). Importantly however, the monosialylated glycoforms in GP18 comprise almost completely of human FA2G2S1 and it is therefore difficult to quantify murine monosialylated glycostructure by HILIC-UPLC-FLR. This prompted us to perform further studies.

### Detection of B Cell Extrinsic IgG Sialylation by xCGE-LIF

**Figure 5:**
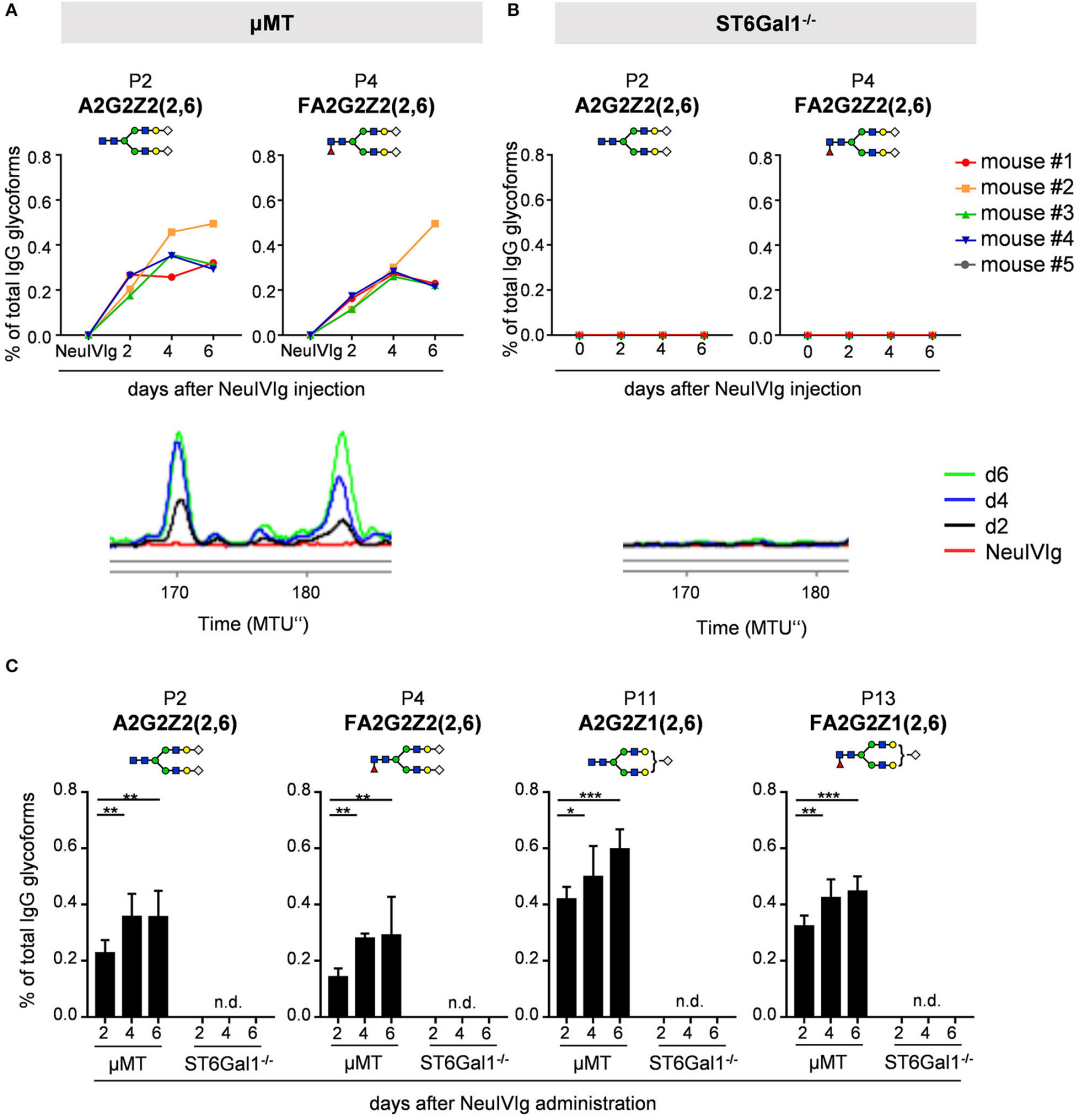
Kinetics of appearance of *de novo* sialylated IgG glycoforms as detected with xCGE-LIF. **(A,B)** Shown are the relative changes (% of total IgG glycoforms in the preparation) in glycopeaks (P) 2 and 4 in neuraminidase treated IVIg (NeuIVIg) before and 2, 4, and 6 days after injection into μMT **(A)** or ST6Gal1 deficient mice **(B)** by xCGE-LIF analysis for individual mice. In the bottom of each Figure a representative xCGE-LIF trace of glycopeaks P2 and 4 is depicted. **(C)** Quantification of all sialylated glycan peaks P2, P4, P11, and P13 2, 4, and 6 days after injection of NeuIVIg into μMT or ST6Gal1^−/−^ mice, respectively. Bars represent mean ± SD. *p = 0.05, **p = 0.01, ***p = 0.001 by repeated measures two-way ANOVA. *n* = 4–5. In each Figure, a schematic drawing of the sugar structure for the respective glycopeak is depicted. MTU”, normalized migration time units; n.d., not detectable.

To ensure that the inability to detect changes in *de novo* generated monosialylated IgG glycoforms was not due to technical reasons, we decided to use multiplexed capillary gel electrophoresis with laser-induced fluorescence detection (xCGE-LIF) to confirm our results. The advantage of xCGE-LIF is that very small amounts of IgG preparations can be analyzed with high sensitivity allowing better resolution for sialylated species. The characteristic glycan profiles of murine and human IgG preparations by xCGE-LIF as well as the structure and peak assignment are depicted in Figure S4. Based on the fact that neuraminidase digested IVIg allowed the most clear-cut identification of *de novo* IgG sialylation *in vivo*, we focused on NeuIVIg-injected μMT and ST6Gal1^−/−^ mice (Figures 4, 5). As shown for HILIC-UPLC-FLR analysis, additional glycan peaks became detectable on NeuIVIg 6 days after injection in B cell deficient μMT mice, which overlapped with IgG sugar structures selectively present on mouse but not human IgG (Figure 4B). Fully consistent with HILIC-UPLC-FLR, the glycan peaks that appeared *de novo* and were increasing over time were mono- (P11 and P13) and disialylated sugar structures (P2 and P4) with (P4 and P13) or without (P2 and P11) core-fucose (Figures 4, 5). Injection of NeuIVIg into ST6Gal1^−/−^ mice did not lead to any detectable changes in IVIg sialylation *in vivo* (Figures 5B,C). Moreover, agalactosylated (G0), mono-galactosylated (G1), and digalactosylated (G2) IgG glycoforms were not changed over time (Figure S5). Thus, xCGE-LIF analysis allowed a better detection of mono- and disialylated IgG sugar structures.

To exclude that the observed minimal level of extrinsic IgG sialylation is due to the use of human IgG in mice, we performed a similar experiment where we injected 1 mg of the murine TA99-IgG2c antibody—directed against the glycoprotein 75—into B cell deficient μMT mice and analyzed alterations in IgG glycosylation in the serum of these animals 10 min as well as 2, 4, and 6 days after injection by xCGE-LIF. As shown in Figure S6 murine IgG acquired sialic acid residues to a similar extend as human IgG. Again, especially disialylated sugar structures (P2 and P4) were increasing over time. The sugar structure FA2G2Z1 (P13) might co-migrate with the large peak present in initial TA99-mIgG2c antibody (around 250 MTU”) and therefore could not be clearly distinguished. In summary, the data obtained with HILIC-UPLC-FLR and xCGE-LIF analysis suggest that a very small amount of IgG molecules—both human and murine—containing two galactose residues can become modified with one or two sialic acid residues *in vivo* in the absence of B cells.

**Figure 6:**
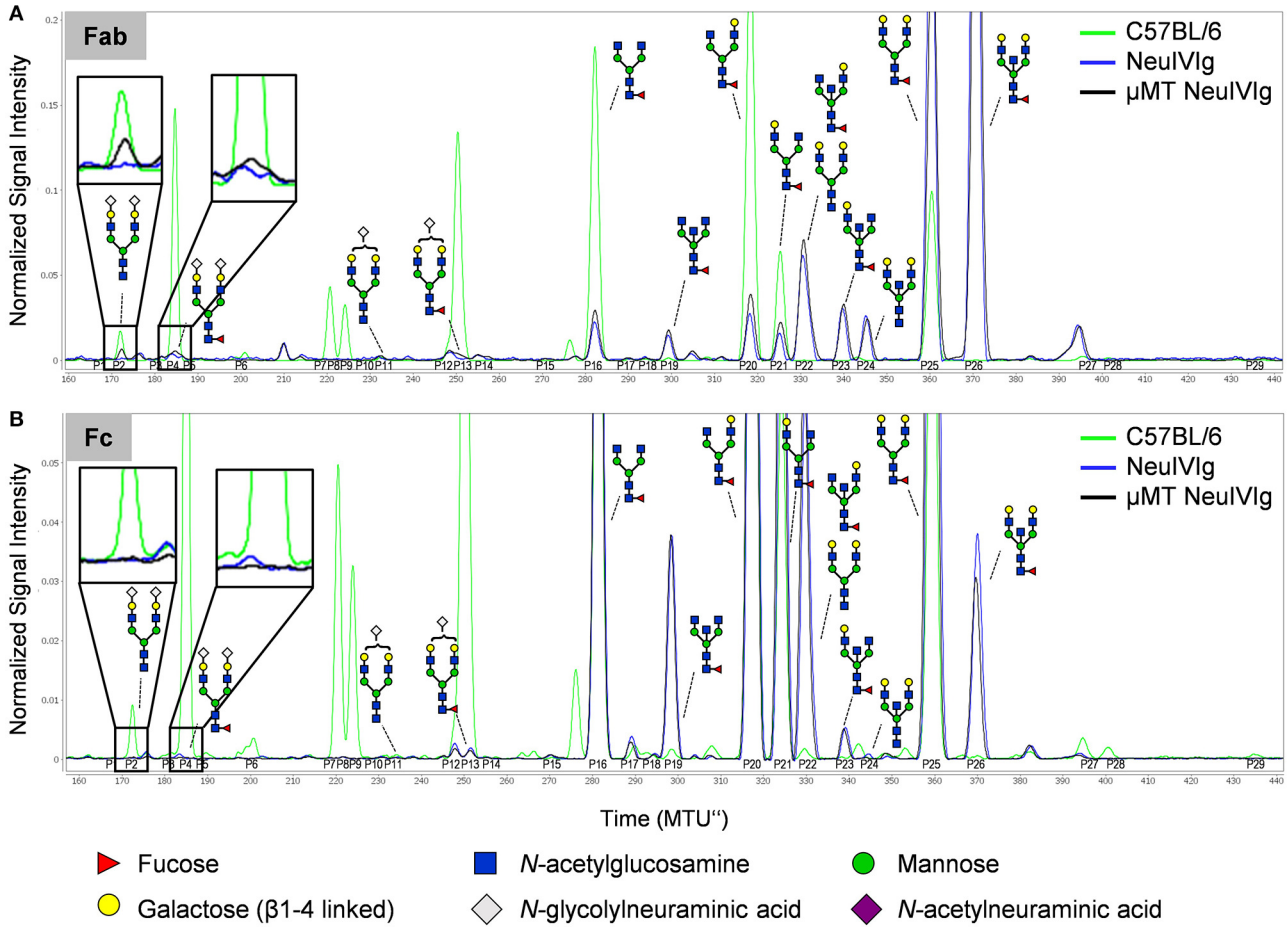
Discrimination of *de novo* sialylation of human IgG *in vivo* between Fab and Fc fragments. **(A,B)** Serum IgG preparations of μMT mice injected with neuraminidase treated IVIg (NeuIVIg) were digested to generate Fab and Fc fragments. To identify *de novo* generated IgG sugar structures on the individual N-glycosylation sites, individual fractions were analyzed separately by xCGE-LIF. Depicted are representative overlays of IgG Fab- **(A)** and Fc-portion **(B)** specific xCGE-LIF glycoanalysis of C57BL/6 mice, the injected NeuIVIg preparation before injection as well as NeuIVIg 4 days after injection into μMT mice. Schematic drawings of the sugar moieties are depicted for the major peaks. Enlarged insets emphasize peaks detected selectively on NeuIVIg 4 days post injection into μMT mice. The Figure legend at the bottom of the Figure depicts the symbols used for individual sugar residues. See also Figure S2 for additional peak information. MTU”, normalized migration time units.

To further characterize extrinsic IgG sialylation, we also discriminated *de novo* generated IgG glycoforms between Fab and Fc. For this purpose, the isolated serum IgG preparations from NeuIVIg injected μMT mice (4 days after NeuIVIg injection) were separated into Fab and Fc portions before glycoanalysis by xCGE-LIF. Individual analysis of the separated IgG fragments revealed that extrinsic IgG sialylation occurred almost exclusively on N-glycosylation sites of the Fab (Figure 6A) but not of the Fc (Figure 6B) fragment. Again, minimal amounts of especially disialylated IgG sugar structures were appearing. In the two mouse serum samples 1.57 ± 0.3% of all IgG glycoforms on the Fab fragment corresponded to disialylated sugar structures, while only 0.89 ± 0.15% were monosialylated. In contrast, no additional murine sialic acids could be detected on the Fc fragment (Figure 6B). Therefore, predominantly the N-linked sugar moieties of the easily accessible Fab fragment in the IVIg preparations seems to be the target for *de novo* sialylation.

### Impact of Extrinsic Sialylation on IgG Effector Function

**Figure 7:**
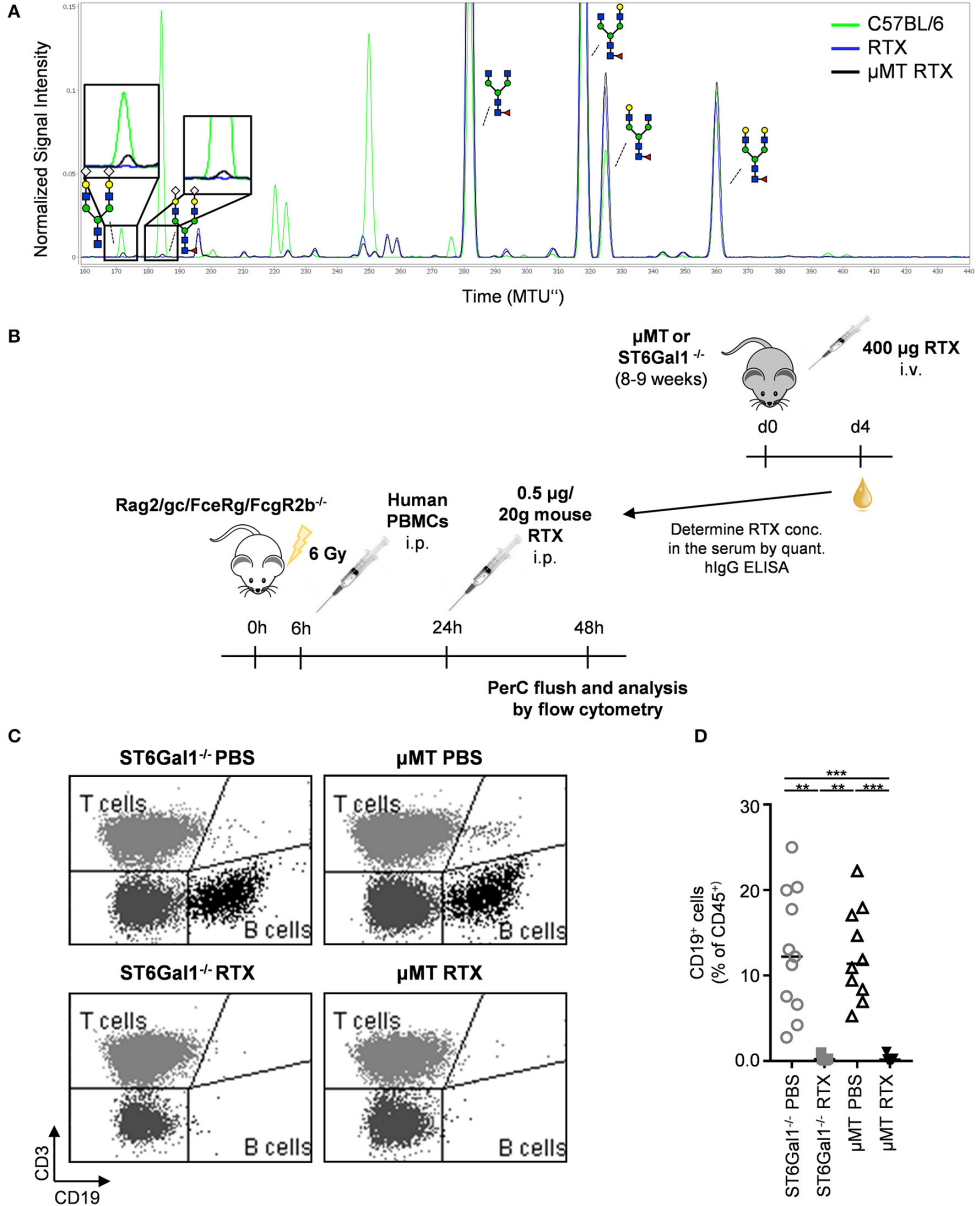
Impact of IgG *de novo* sialylation on cytotoxic antibody activity. Shown is a representative overlay of xCGE-LIF IgG glycoanalysis of rituximab (RTX) treated μMT mice **(A)**, a schematic overview of the experimental setup **(B)**, and its respective results **(C,D)**. **(A)** μMT mice were injected with 400 μg RTX (μMT RTX) and 4 days later serum IgG was analyzed by xCGE-LIF to identify *de novo* generated IgG glycoforms. Schematic drawings of the sugar moieties are depicted for the major peaks. Enlarged insets emphasize peaks detected selectively on RTX 4 days post injection into μMT mice. **(B)** Immunodeficient Rag2/γc/FcεRγ/FcγR2b^−/−^ mice were irradiated (6 Gy) and reconstituted with human PBMC in the peritoneal cavity. Eighteen hours later mice (*n* = 5–6) received equal amounts of rituximab (0.5 μg/20 g mouse) derived from μMT or ST6Gal1^−/−^ mice, which had been injected with rituximab 4 days before. Twenty-four hours after RTX injection, cells of the peritoneal cavity were analyzed by flow cytometry. Shown are representative FACS plots **(C)** and the quantification **(D)** of human B cell counts in the peritoneal cavity 1 day after rituximab or PBS injection. Statistical significance was evaluated with a Kruskal-Wallis test followed by Dunns *post-hoc* test. **p = 0.01, ***p = 0.001. Horizontal lines represent median values.

To evaluate the impact of extrinsic sialylation on cytotoxic IgG effector functions, we injected μMT and ST6Gal1^−/−^ mice with the CD20 specific antibody rituximab, which is broadly used in patients with autoimmune diseases and cancer. As rituximab selectively recognizes human but not mouse CD20, the injection into ST6Gal1 deficient mice with Rituximab does not affect B cell numbers. Four days after injection, serum from μMT and ST6Gal1 knockout mice was collected, the level of human IgG determined and extrinsic IgG sialylation assessed by xCGE-LIF. As shown in Figure 7A, very low amounts of exclusively disialylated IgG structures were found in rituximab injected μMT mice. To assess the functional activity of human CD20 antibodies present in the serum of μMT and ST6Gal1 deficient mice, a serum equivalent containing 0.5 μg rituximab was injected into immunodeficient Rag2/γc/FcεRγ/FcγR2b^−/−^ mice, which were irradiated and reconstituted with human peripheral blood mononuclear cells 18 h before (Figure 7B). As shown in Figures 7C,D, rituximab treatment via serum transfer of rituximab-injected μMT and ST6Gal1^−/−^ mice led to a significant depletion of B cells in the peritoneal cavity, while B cell counts in PBS-serum treated mice were not affected. In line with the low efficiency of the extrinsic sialylation pathway, when we compared B cell depletion between rituximab-containing ST6Gal1^−/−^ and μMT serum, no significant differences in cytotoxic antibody activity were detected.

## Discussion

An important step in the process of introducing a new therapeutic IgG antibody or a new batch of an already approved antibody into the clinic is an in-depth characterization of the IgG glycovariants present in the antibody preparation. It is well-known that many factors, such as the cell line in which a therapeutic antibody is produced (43) or the specific culture conditions (44), can alter IgG glycosylation. The importance of detecting alterations in IgG glycosylation have been emphasized by the fact that relatively minor changes in the composition of the biantennary complex sugar structure attached to both of the IgG Fc-domains can dramatically alter IgG activity. Thus, IgG antibodies lacking fucose, sialic acid, and galactose have been described to have an enhanced pro-inflammatory activity, while antibody glycoforms rich in terminal sialic acid residues have an active anti-inflammatory and immunomodulatory activity (5, 14, 45, 46). Until recently, it was believed that IgG glycosylation is established exclusively within the cells in which the antibody is produced and remains rather stable upon injection *in vivo*. However, more recent evidence suggests that IgG glycosylation may be actively altered *in vivo* (20, 21, 47). Thus, IgG antibodies were described to become sialylated post secretion from plasma cells. As enhanced IgG sialylation was shown to impact IgG activity, this would represent a major concern for the use of therapeutic antibodies in patients, prompting us to analyze to what extent this extrinsic IgG sialylation pathway affects passively transferred antibodies. Technically this represents a major challenge as *in vivo* several factors may lead to an altered abundance in IgG glycoforms over time. For example, a more rapid clearance of select IgG isotypes or glycoforms may lead to an increase in other IgG glycoforms with a longer IgG half-life (41, 48). In a similar manner, using mouse strains with a cell subset specific deletion of ST6Gal1, the enzyme that catalyzes the addition of α2,6-linked terminal sialic acid residues on IgG antibodies, may overestimate the level of B cell extrinsic sialylation if the deletion in the target cell population is incomplete and if B cell subsets that have escaped ST6Gal1 deletion have a competitive advantage over those with a deletion of the enzyme.

To study this in a most informative setting we decided to analyze changes in human IgG sialylation in mice lacking mature B cells and serum antibodies. To get the most complete picture we used human intravenous immunoglobin preparations (IVIg), as IVIg contains all human IgG subclasses present in serum. In this experimental scenario, human IgG molecules represent the only antibody isotype in the serum and hence should be fully accessible for *de novo* sialylation. Moreover, mouse terminal sialic acid residues (**Neu5Gc**) can be distinguished from human sialic acid residues (**Neu5Ac**) by analytical techniques allowing an unequivocal identification of newly added mouse derived sialic acid residues to the transferred human antibodies *in vivo* (38). By using two independent analytical techniques to assess extrinsic *de novo* IgG sialylation *in vivo*, our results suggest that the addition of sialic acid residues is a very rare, but nonetheless detectable event. Interestingly the acceptor sugar moieties accessible for the *de novo* mono- or disialylation seemed to contain two terminal galactose residues (G2 glycoform), while in principle also monogalactosylated (G1) glycoforms could have served as acceptor structures for *de novo* sialylation and are normally present in mice *in vivo*. Moreover, both core fucosylated G2 and non-fucosylated G2 sugar moieties were able to acquire terminal Neu5Gc. Of note, increasing the amount of potential acceptor sugar structures by pre-treating human IgG with neuraminidase only marginally increased the level of IgG sialylation, further strengthening the notion, that extrinsic IgG sialylation is a rather inefficient process. However, it has to be considered that also the monosialylated structures as potential acceptor structures were removed. More importantly, when IgG preparations such as IVIg were used, in which IgG molecules present additionally containing N-linked sugar moieties in the Fab portion as potential acceptor sites for extrinsic sialylation, an almost exclusive sialylation occurred in these more easily accessible sugar domains (although not at a higher efficacy). As Fab arm glycosylation may modify antibody specificity, it will be interesting to study if extrinsic Fab arm glycosylation affects target antigen binding (49). If no Fab associated sugar moieties were present, however, minor changes in Fc-linked sugar moieties could be detected for mouse and human monoclonal antibodies. As the extent of B cell extrinsic IgG sialylation was similar for mouse and human IgG, we would exclude that human IgG molecules become sialylated less efficiently in mice due to some species barrier effects. Furthermore, as the amount of *de novo* sialylated IgG never exceeded two percent of the total IgG present *in vivo*, one would not expect functional consequences for IgG activity. Indeed, the human CD20 specific antibody Rituximab did not show an altered activity when passaged through B cell deficient mice.

In summary, our study demonstrates that the process of B cell extrinsic *de novo* sialylation of IgG antibodies *in vivo* is only affecting a minor subset in the pool of IgG glycovariants present in IVIg and cytotoxic IgG preparations and hence may not trigger altered pro- or anti-inflammatory IgG activities.

## Data Availability Statement

The datasets generated for this study are available on request to the corresponding author.

## Ethics Statement

The animal study was reviewed and approved by the government of Lower Franconia.

## Author Contributions

AS designed, performed, and analyzed the experiments. MH, MN, OZ, and JK analyzed the samples. RH and ER developed the method and software for xCGE-LIF analysis. AS and FN wrote the paper. AL, LN, and MPei provided material. MH, OZ, JK, AL, MPez, GL, MPei, and LN discussed the data. All authors read, reworked, and approved the manuscript.

### Conflict of Interest

GL is the founder and CEO of Genos—a private research organization that specializes in high-throughput glycomic analysis and has several patents in the field. MH, MN, OZ, JK, and MPez are employees of Genos. ER is founder, CEO and CSO of glyXera GmbH and RE is an employee of glyXera GmbH. glyXera provides high-throughput glycomic analysis and holds several patents for xCGE-LIF based glycoanalysis. The remaining authors declare that the research was conducted in the absence of any commercial or financial relationships that could be construed as a potential conflict of interest.
