# Supplementary material for: Minimal B Cell Extrinsic IgG Glycan Modifications of Pro- and Anti-Inflammatory IgG Preparations in vivo
Source: Front Immunol. 2020 Jan 9;10:3024. doi: 10.3389/fimmu.2019.03024 (PMC6970187; doi:10.3389/fimmu.2019.03024)
Supplement: Supplementary file 1 [file Data_Sheet_1.pdf]

Supplementary Material

Figure S1

A

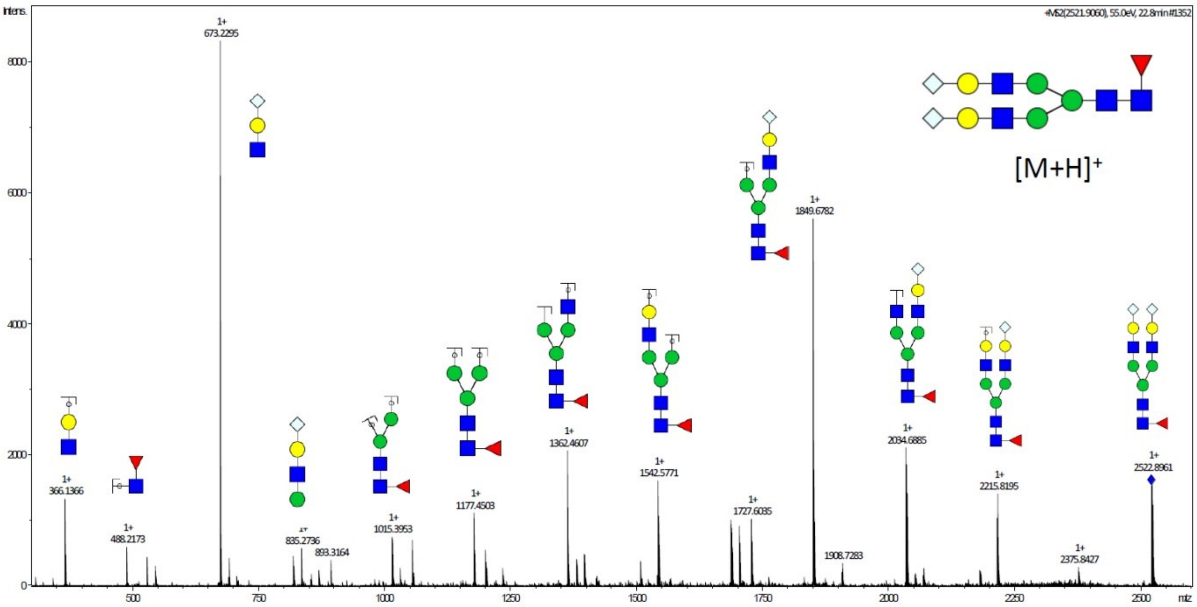

B

UPLC Peak #	M	[M+H]$^{+}$	[M+2H]$^{2+}$	Composition	Proposed structure	Comment
**GP18**	2067,67	2068,67	1034,8342	(Hex)2 (HexNAc)2 (NeuGc)1 + (Man)3(GlcNAc)2	**A2G2Z1**	Major composition
	2213,73	2214,73	1108,36	(Hex)2 (HexNAc)2 (Deoxyhexose)1 (NeuGc)1 + (Man)3(GlcNAc)2	**FA2G2Z1**	Minor composition
**GP20**	2213,73	2214,73	1108,36	(Hex)2 (HexNAc)2 (Deoxyhexose)1 (NeuGc)1 + (Man)3(GlcNAc)2	**FA2G2Z1**	Major composition
**GP21**	2416,87	2417,87	1209,43	(Hex)2 (HexNAc)2 (NeuGc)2 (Ac)1 + (Man)3(GlcNAc)2	**A2G2Z2Ac1**	Mix of compositions
				(Hex)2 (HexNAc)3 (NeuGc)1 (Deoxyhexose)1 + (Man)3(GlcNAc)2	**FA2BG2Z1**	
**GP23**	2375,87	2376,87	1188,93	(Hex)3 (HexNAc)2 (Deoxyhexose)1 (NeuGc)1 + (Man)3(GlcNAc)2	**FA2G3Z1**	Major composition
	2488,91	2489,91	1245,46	(Hex)2 (HexNAc)2 (Deoxyhexose)1 (NeuAc)2 + (Man)3(GlcNAc)2	**FA2G2S2**	Major composition
**GP24**	2520,91	2521,91	1261,45	(Hex)2 (HexNAc)2 (Deoxyhexose)1 (NeuGc)2 + (Man)3(GlcNAc)2	**FA2G2Z2**	Minor composition
	2374,84	2375,84	1188,42	(Hex)2 (HexNAc)2 (NeuGc)2 + (Man)3(GlcNAc)2	**A2G2Z2**	Major composition
**GP25**	2504,91	2505,91	1253,46	(Hex)2 (HexNAc)2 (Deoxyhexose)1 (NeuAc)1 (NeuGc)1 + (Man)3(GlcNAc)2	**A2G2Z1S1**	Major composition
**GP25**	2374,84	2375,84	1188,42	(Hex)2 (HexNAc)2 (NeuGc)2 + (Man)3(GlcNAc)2	**A2G2Z2**	Major composition
	2520,91	2521,91	1261,45	(Hex)2 (HexNAc)2 (Deoxyhexose)1 (NeuGc)2 + (Man)3(GlcNAc)2	**FA2G2Z2**	Minor composition
**GP26**	2520,91	2521,91	1261,45	(Hex)2 (HexNAc)2 (Deoxyhexose)1 (NeuGc)2 + (Man)3(GlcNAc)2	**FA2G2Z2**	Major composition

Figure S1: Confirmation of sialylated glycopeak structures by LC-MS

The structures of the indicated glycopeaks (GP) were confirmed by LC-MS (more precisely, by HILIC-UPLC-ESI-QTOF-MS/MS). (A) An exemplary fragmentation spectrum of GP26 (FA2G2Z2) is shown including an assignment of schematic drawings of the sugar structures for the major peaks (A). (B) Table summarizing the mass spectrometric analysis of GPs corresponding to sialylated glycan structures (GP18, 20, 21, and GP23-26). Calculated mass and *m/z*-ratios of singly ($[M+H]^{+}$) and doubly charged ($[M+2H]^{2+}$) glycan species, composition, proposed structure and respective comments are listed for each GP. Structure abbreviations: F, a core fucose α1–6 linked to the inner N-acetylglucosamine (GlcNAc); Ax, number of antenna (GlcNAc); B, bisecting GlcNAc linked β1–4 to mannose; Gx, number of β1–4 linked galactose (G) on antenna; αGx, number of α1–3 linked galactose linked to β1–4 galactose; Sx, number of N-acetylneuraminic acids (S) linked to β1–4 galactose; Zx, number of N-glycolylneuraminic acids (Z) linked to β1–4 galactose; Ac, O-Acetylation.

Figure S2

A

Peak	Annotation (human)	Annotation (mouse)	Structure (human)	Structure (mouse)
1	FA1	FA1		
2	A2	n.d.		
3	A2B	n.d.		
4	FA2	FA2		
5	Man5	n.d.		
6	FA2B<br>A2G1[6]	FA2B		
7	A2G1[3]	n.d.		
8	FA2G1[6]<br>A2BG1	FA2G1[6]		
9	FA2G1[3]	FA2G1[3]		
10	FA2BG1[6]	FA2BG1[6]		
11	FA2BG1[3]	n.d.		
12	A2G2	n.d.		
13	A2BG2	n.d.		
14	FA2G2	FA2G2		
15	FA2BG2<br>FA1G1S1<br>A2G1S1	n.d.		
16	FA2G1[6]S1<br>FA2G1[3]S1	n.d.		
17a	A2G2S1	FA2G1[6]Z1		
17b		FA2G1[3]Z1		
18	A2BG2S1<br>FA2G2S1	A2G2Z1<br>FA2G2Z1		
19	FA2BG2S1	n.d.		
20	n.d.	FA2G2Z1		
21	A2G2S2	A2G2Z2Ac1<br>FA2BG2Z1		
22	A2BG2S2	n.d.		
23	FA2G2S2	FA2G2aG1Z1		
24	FA2BG2	FA2G2Z2<br>A2G2Z2		
25	x	A2G2Z1S1<br>FA2G2Z2<br>A2G2Z2		
26	x	FA2G2Z2		

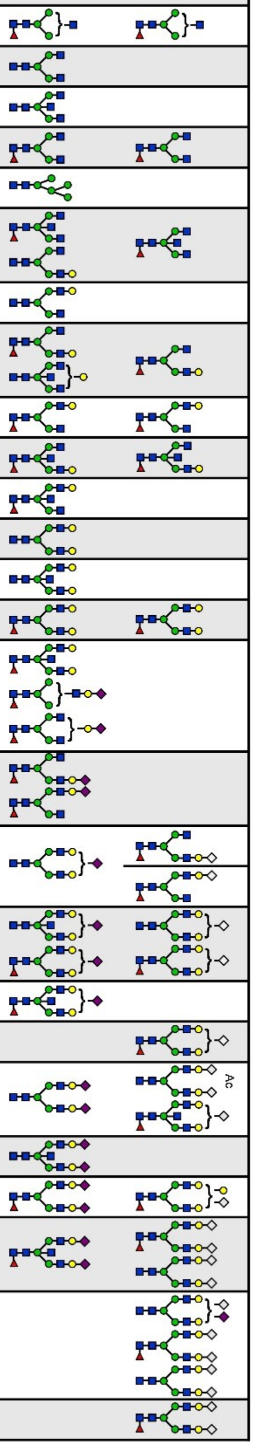

B

Peak	Annotation	Structure
1	A2G2S2(2,6)	
2	A2G2Z2(2,6)	
3	FA2G2S2(2,6)	
4	FA2G2Z2(2,6)	
5	FA2BG2S2(2,6)	
6	A2G1S1(2,6)[6]	
7	FA2G1S1(2,6)[6]	
8	FA2G1S1(2,6)[3]	
9	A2G2S1(2,6)[6]	
10	A2G2S1(2,6)[3]	
11	A2G2Z1(2,6)	
12	FA2G2S1(2,6)<br>Man5	
13	FA2G2Z1(2,6)	
14	FA2BG2S1<br>A2	
15	A2B	
16	FA2	
17	A2G1[6]	
18	A2G1[3]	
19	FA2B	
20	FA2G1[6]	
21	FA2G1[3]	
22	FA2BG1[6]<br>A2G2	
23	FA2BG1[3]	
24	A2BG2	
25	FA2G2	
26	FA2BG2	
27	FA2G2αG1[6]	
28	FA2G2αG1[3]	
29	FA2G2αG2	

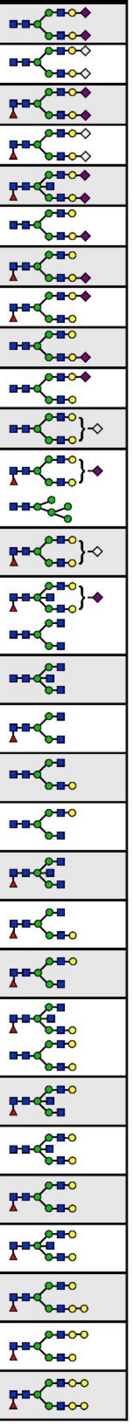

Fucose — *N*-acetylglucosamine — Mannose

Galactose (β1-4 linked) — *N*-glycolylneuraminic acid — *N*-acetylneuraminic acid

Figure S2: Nomenclature, annotation and structure of different glycan peaks

Depicted are the annotations and structures of each glycopeak obtained by HILIC-UPLC-FLR (A) and xCGE-LIF (B) analysis. For HILIC-UPLC-FLR annotation and structure is discriminated between human and mouse IgG glycoforms. Schematic drawings of the sugar structures for the respective peaks are depicted. The Figure legend at the bottom of the Figure depicts the symbols used for individual sugar residues. Structure abbreviations: F, a core fucose α1–6 linked to the inner N-acetylglucosamine (GlcNAc); Ax, number of antenna (GlcNAc); B, bisecting GlcNAc linked β1–4 to mannose; Gx, number of β1–4 linked galactose (G) on antenna; G1[3] and G1[6] indicates that the galactose is on the antenna of the α1–3 or α1–6 mannose; αGx, number of α1-3 linked galactose linked to β1–4 galactose; Sx, number of N-acetylneuraminic acids (S) linked to β1–4 galactose; Zx, number of N-glycolylneuraminic acids (Z) linked to β1–4 galactose; Ac, O-Acetylation.

Figure S3

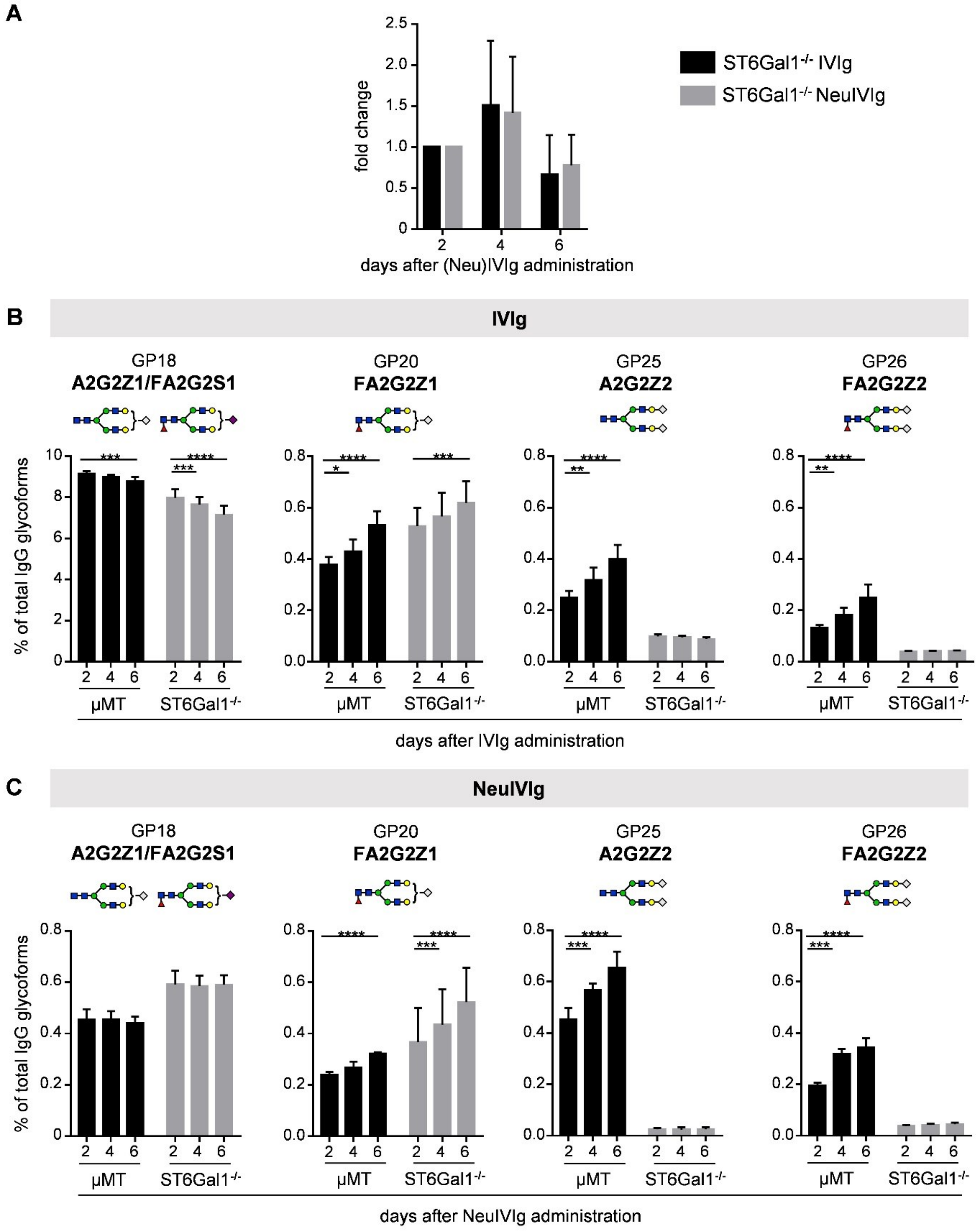
A
fold change
2.5
2.0
1.5
1.0
0.5
0
2
4
6
days after (Neu)IVIg administration
ST6Gal1-/- IVIg
ST6Gal1-/- NeuIVIg
B
IVIg
GP18
A2G2Z1/FA2G2S1
GP20
FA2G2Z1
GP25
A2G2Z2
GP26
FA2G2Z2
% of total IgG glycoforms
μMT
ST6Gal1-/-
days after IVIg administration
C
NeuIVIg
GP18
A2G2Z1/FA2G2S1
GP20
FA2G2Z1
GP25
A2G2Z2
GP26
FA2G2Z2
% of total IgG glycoforms
μMT
ST6Gal1-/-
days after NeuIVIg administration

Figure S3: IVIg antibody half-life and kinetics of appearance of de novo sialylated IgG glycoforms as obtained by HILIC-UPLC-FLR

(A) Depicted is the fold change of human IgG concentrations in the sera of IVIg or neuraminidase treated IVIg (NeuIVIg) injected ST6Gal1$^{-/-}$ mice at the indicated time points. (B, C) Shown are the relative changes (% of total IgG glycoforms in the preparation) in glycopeaks (GP) 18, 20, 25 and 26 of IVIg (B) or neuraminidase treated IVIg (C) two, four, and six days after injection into µMT and ST6Gal1 deficient mice by HILIC-UPLC-FLR analysis. Bars represent mean ± SD. *p=0.05, **p=0.01. ***p=0.001, ****p=0.0001 by repeated measures two-way ANOVA. n=4-5. In each Figure, a schematic drawing of the sugar structure for the respective glycopeak is depicted.

Figure S4

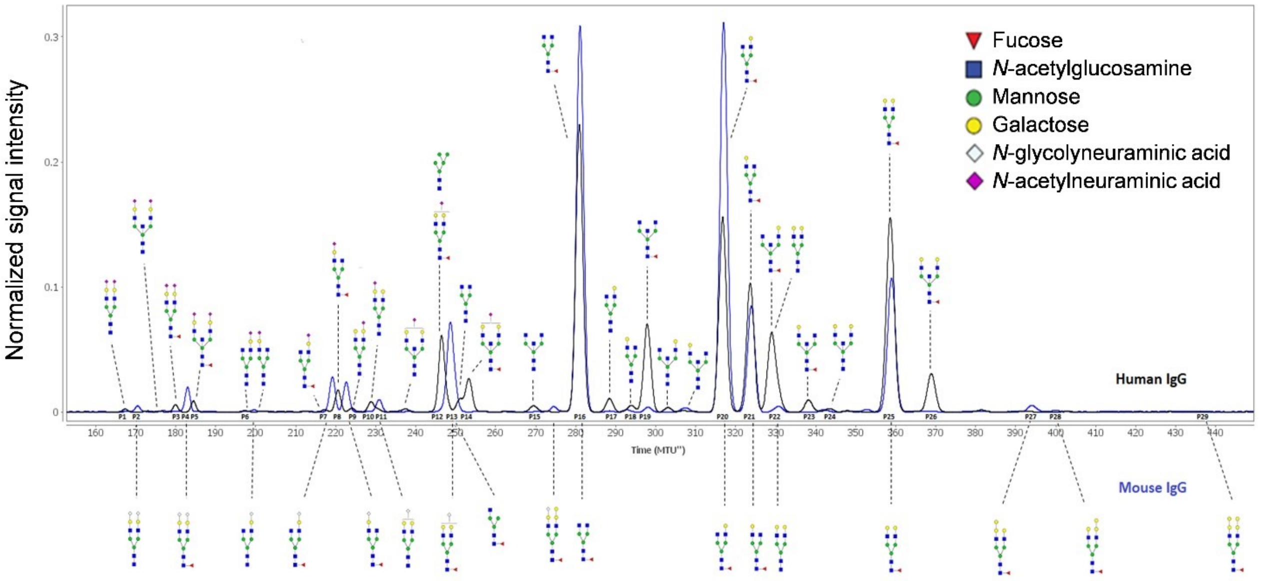

Figure S4: Representative murine and human IgG glycan profile detected with xCGE-LIF analysis

Shown are representative chromatograms of murine (blue) and human (black) IgG glycan profiles obtained by xCGE-LIF analysis. Schematic drawing of the sugar structure for each individual glycopeak (P1 – P29) is depicted (human structures on top, mouse structures on the bottom). The Figure legend at the upper right of the Figure depicts the symbols used for individual sugar residues.

Figure S5

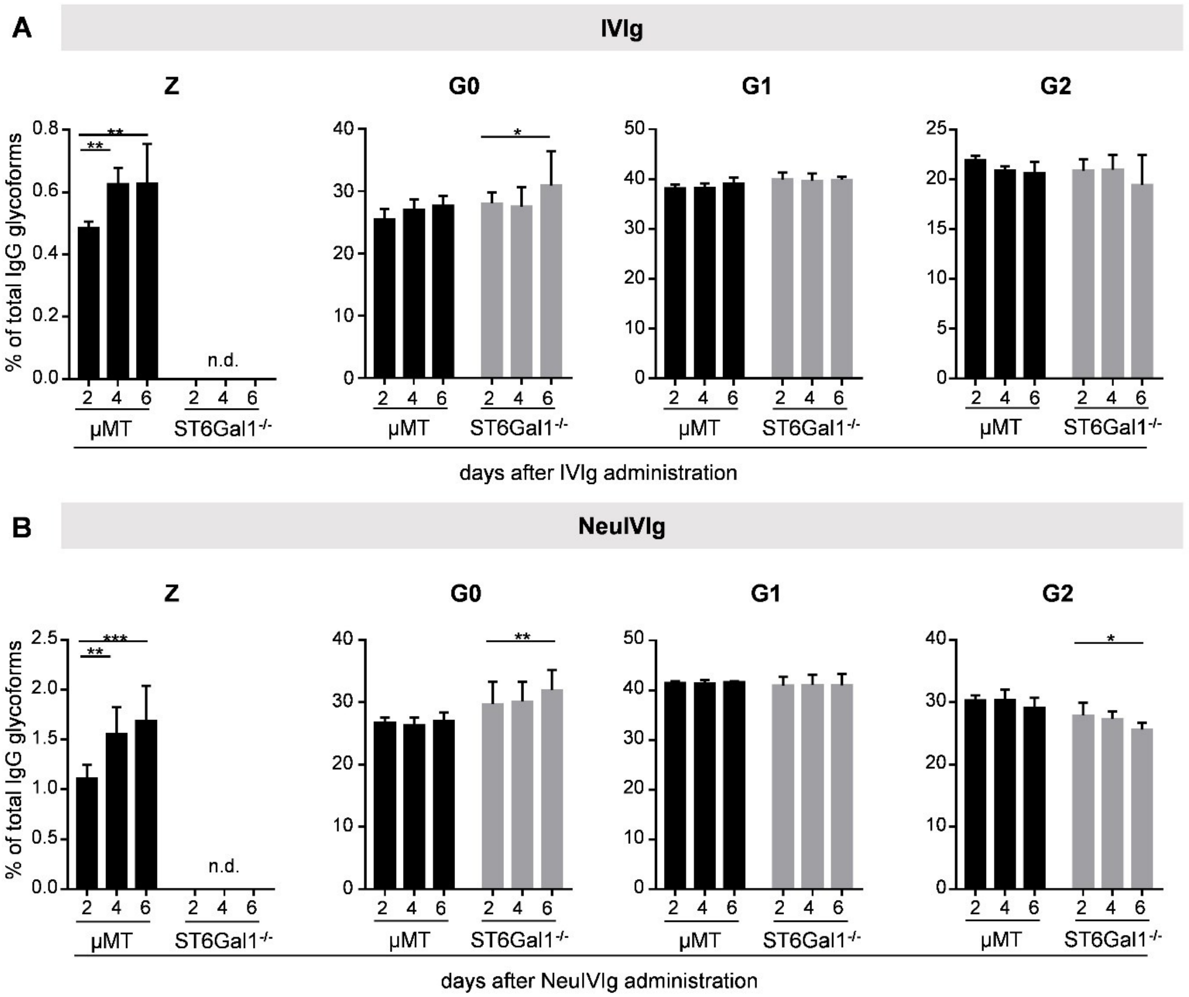

Figure S5: Kinetics of different IgG glycoforms as obtained by xCGE-LIF analysis.

(A, B) Shown are the relative changes (% of total IgG glycoforms in the preparation) of the sialylated (Z), agalactosylated (G0), monogalactosylated (G1) and digalactosylated (G2) glycoforms of IVIg (A) or neuraminidase treated IVIg (B) two, four, and six days after injection into µMT and ST6Gal1 deficient mice by xCGE-LIF analysis. Bars represent mean ± SD. *p=0.05, **p=0.01. ***p=0.001, by repeated measures two-way ANOVA. n=4-5. n.d.: not detectable.

Figure S6

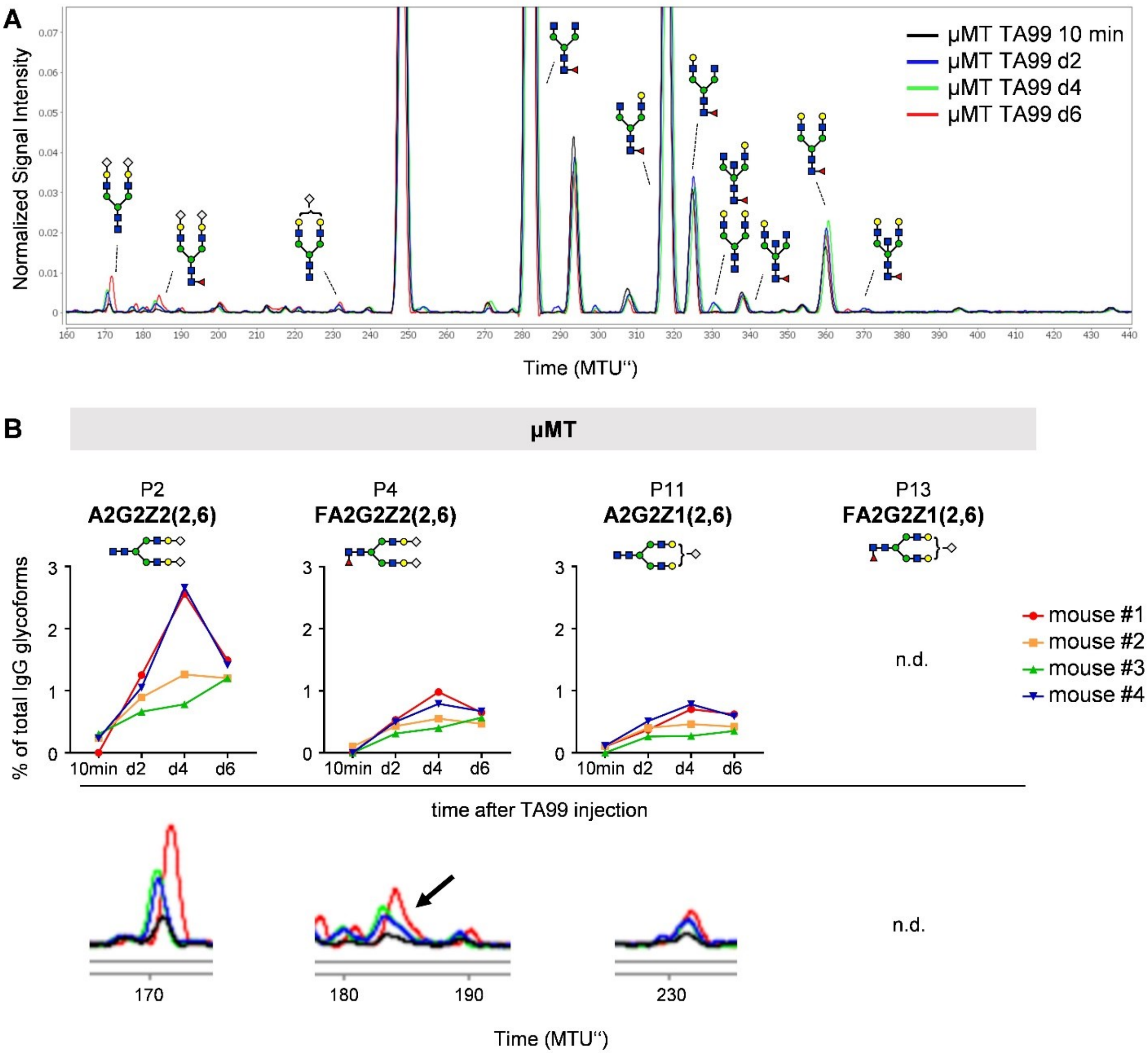

Figure S6: Kinetics of appearance of de novo sialylated IgG glycoforms on murine IgG.

(A) Shown is a representative overlay of xCGE-LIF IgG glycoanalysis of TA99 ten minutes as well as two, four and six days after injection into µMT mice (µMT TA99 10 min/d2/d4/d6). Clearly identifiable glycan peaks are annotated with their respective schematic drawing of the sugar structure. (B) Shown are the relative changes (% of total IgG glycoforms in the preparation) in glycopeaks (P) 2, 4 and 11 of TA99 ten minutes as well as two, four, and six days after injection into µMT mice by xCGE-LIF analysis for individual mice (n=4). In the bottom of each Figure a representative xCGE-LIF trace of glycopeaks P2, 4 and 11 is depicted (colors of individual traces correspond to Fig. S6A). P13 was not clearly identifiable and therefore was not determined (n.d.). In each Figure a schematic drawing of the sugar structure for the respective glycopeak is shown. Abbreviations: MTU'': normalized migration time units.
